# Emotion Recognition and the Screening Instrument for Borderline Personality Disorder (SI-Bord): Outcomes and Community-Based Validation

**DOI:** 10.3390/brainsci12111512

**Published:** 2022-11-08

**Authors:** Emmet Godfrey, Molly Kelly Grealy, Erin Whyte O’Sullivan, Sarah Sullivan, Finn Brady, Grace Carroll, Tom Burke

**Affiliations:** 1 School of Psychology, National University of Ireland Galway, H91 TK33 Galway, Ireland; 2 Department of Psychology, University of Notre Dame, Notre Dame, IN 46556, USA; 3 School of Psychology, Queen’s University Belfast, Belfast BT9 7BL, UK

**Keywords:** borderline personality disorder, SI-Bord, social cognition, screening validation

## Abstract

Background: Borderline Personality Disorder (BPD) is a psychiatric condition characterized by impulsivity, affect instability, dysregulation, low self-image, and interpersonal difficulties. There are many instruments to measure traits of BPD, however, few can be administered quickly. The Screening Instrument for Borderline Personality Disorder (SI-Bord) is an instrument offering a brief administration time with comparable psychometric properties to more comprehensive measures. The present study aimed to evaluate the psychometric properties of the SI-Bord in a healthy community-based sample and its relatedness to measures of social cognition. Methods: A community-based sample of participants completed an online survey consisting of measures of BPD traits and social cognition including: the Screening Instrument for Borderline Personality Disorder (SI-Bord), the Personality Assessment Inventory (PAI), the Florida Affect Battery (FAB), the Interpersonal Reactivity Index (IRI), and the Narcissistic Personality Inventory (NPI). Reliability was assessed using Cronbach’s alpha and inter-item correlations. Validity was assessed using factor analysis, examining associations with other measures of BPD traits, and examining associations with measures not measuring BPD traits. Results: 151 participants were included in the study. Participants’ age ranged from 20–76 (mean age of 38.79 ± 12.37) and comprised 76 females (50.33%) and 75 males (49.67%). Good internal consistency was found with a Cronbach’s alpha of 0.71. Good inter-item reliability was found with a mean inter-item cross correlation of 0.25, with each item of the SI-Bord showing an inter-item correlation coefficient of >0.5. Factor analysis identified good construct validity with a strong singular dimension explaining a large proportion of variance (Question 1). The SI-Bord showed good concurrent validity with significantly strong positive correlations with the subscales of the PAI borderline scale measuring affect instability (r = 0.60; *p* < 0.001), identity problems (r = 0.67; *p* < 0.001), negative relationships (r = 0.61; *p* < 0.001), total score (r = 0.76; *p* < 0.001), and to a moderately strong positive correlation with self-harm (r = 0.39; *p* < 0.001). The SI-Bord was not correlated with the NPI-16 (r = 0.131; *p* = 0.11), showing good divergent validity. Conclusions: These findings support the SI-Bord as a quick and useful screening tool for traits associated with BPD. Further clinical validation is warranted.

## 1. Introduction

Borderline Personality Disorder (BPD) is a psychiatric condition characterized by a pervasive pattern of marked impulsivity, affect instability or dysregulation, low self-image, and interpersonal relationship difficulties [1]. BPD is a prominent clinical disorder effecting 5.9% of the general population [2], 11% of psychiatric outpatients [3] and 33% of inpatients in mental health settings [4], with an increasing incidence [5].

The diagnostic criteria outlined by the Diagnostic and Statistical Manual of Mental Disorders (DSM-V), requires an individual’s particular symptoms of ‘maladaptive personality traits’ to be pervasive, persistent, and unlikely to be limited to a particular developmental stage or another mental disorder [1]. Symptoms of BPD relate to the severity of identity problems, self-direction, empathy, and intimacy, as well as the presence of at least four out of seven ‘pathological’ personality traits. According to the American Psychological Association (APA), these traits include emotional lability, anxiety, separation distress, depression, impulsivity, unhealthy risk-taking behaviour, and hostility [1]. While BPD shares symptom-level characteristics with a range of other personality disorders, early life adversity and difficulties with social functioning, even at the “healthiest level of functioning” are reported to be distinguishable features of BPD, relative to other disorders [6].

Clinical and empirical observations have proposed that impaired social cognition is a mechanism underlying the maintenance of features of BPD [7]. Social cognition is a cognitive domain that includes comprehending others’ intentions, beliefs, feelings, and mental states, as well as social interaction, social context, empathy, and social decision making [8,9,10,11]. Historically, it has been shown that people with a clinical diagnosis of BPD show impaired social relatedness [12] and interpret more malevolent representations of people’s intentions and actions [13]. A more recent study further showed that BPD patients evaluated characters from silent film clips as more negative and more aggressive compared to depressed and non-clinical controls [14].

The breadth of measures used in the assessment of BPD is varied, with Meaney and colleagues [15] finding 13 assessment tools across 133 articles in a systematic review of prevalence of BPD in university samples. The most widely used instruments were found to be the Structured Clinical Interview for DSM-III-R/DSM-IV (SCID-II), the Personality Assessment Inventory-Borderline Scale (PAI-BOR), and the McLean Screening Instrument for Borderline Personality Disorder. Wongpakaran and colleagues [16] then developed the short screening tool for BPD traits, known as the Short-Bord, while maintaining the psychometric properties of longer instruments. The key items were Items 1 (instability when abandoned), 2 (unstable relationships), 3 (sudden identity changes), 8 (self-harm or suicidal tendencies), and 10 (unstable mood). These are the 5 items used in the Short-Bord. Lohanan and colleagues [17] then developed the Screening Instrument for Borderline Personality Disorder (SI-Bord). This revised version of the Short Bord aimed to increase reliability and validity. The SI-Bord utilizes the same 5 key items determined in the Short-Bord. However, instead of dichotomous true/false responses, the SI-Bord used a 5-point Likert scale. The psychometric properties of the SI-Bord were found to be valid and reliable, based on a sample of university students. This measure represents an alternative to the widely used measures mentioned above. Due to its briefness, it offers simplicity to administer, score, and interpret, which at the screening stage of assessment may be valuable for clinical utility. Furthermore, each of the SI-Bord constructs relate to known symptom clusters associated with BPD [1,6], and so the SI-Bord specifically may provide useful single-item scales for investigating specific BPD traits in research. If found to be a reliable scale with a non-university population, this could increase the accessibility of trait-based features of BPD in research and reduce the administration time. To date, no studies have examined the validity and reliability of the SI-Bord in a non-university student population, nor its relatedness to performance on measures of social cognition. Consequently, the aim of this study was to investigate the psychometric properties of the SI-Bord in a group of community-based healthy participants and investigate its relatedness to measures of social cognition.

## 2. Materials and Methods

### 2.1. Participants and Procedure

This study employed a cross-sectional survey-based design from a community-based sample of typical controls. Data from 151 participants were gathered using Prolific, an online platform for survey-based data collection (www.prolific.com; last accessed on 31 November 2022). Participants were required to be over the age of 18, to give explicit consent, and to be residents in the Republic of Ireland. Exclusion criteria included having existing neurological or mental health diagnoses which may interfere with test performance and being non-native English speakers. Only participants who met the above criteria as determined by their Prolific profile, were invited to engage with the study. Participants were required to confirm their eligibility, in terms of each of the inclusion and exclusion criteria, prior to commencing the study. Participants were informed that only fully finished surveys with >95% response rates would be considered complete; this is to allow a participant to omit a question should they wish due to uncomfortableness in responding, without leading to missing data through survey-based responses. On completion of the survey, participants received a gratuity commensurate with the hourly minimum wage rate in Ireland. A pilot study was conducted with 10 participants to ascertain acceptability and feasibility, as well as expected duration of questionnaire completion, with no changes made following this. Consequently, the study was continued and the data from these 10 was retained. The average duration of the experiment was approximately 25 min. The School of Psychology National University of Ireland Galway Health Research Ethics Committee approved this study. The participants provided explicit informed consent to participate in this study. All procedures were conducted in accordance with the principles expressed in the Declaration of Helsinki.

### 2.2. Measures

(1) The Screening Instrument for Borderline Personality Disorder (SI-Bord)

As above, the Screening Instrument for Borderline Personality Disorder (SI-Bord) [17] consists of a 5-item self-report questionnaire on the key features of BPD from the DSM-5; abandonment avoidance, interpersonal relationship instability, identity disturbance, suicidal and self-harm behaviours, and affective instability. The measure uses a 4-point Likert scale ranging from (0) “Not at all”, (1) “A little”, (2) “Somewhat”, to “to a great extent” (3). The SI-Bord has adequate discriminative power between cases and non-cases of BPD and Cronbach’s alpha indicated acceptable internal consistency within a student population (0.76) [17]. The SI-Bord is satisfactory in its diagnostic accuracy, which is comparable to other vetted screening questionnaires with an Area Under the Receiver Operating Characteristics Curve (AUC) of 0.83. This AUC figure indicates there is an 83% chance that the SI-Bord will correctly distinguish BPD cases from non-BPD cases. Furthermore, intraclass correlation analysis yielded a coefficient of 0.925, which is considered excellent.

(2) The Florida Affect Battery

The Florida Affect Battery (FAB) [18], is a measure of emotion recognition, designed to assess the perception of facial and prosodic affect under a variety of task demands. Five different emotions are used across the subtests: happiness, sadness, anger, fear, and neutral. Subtests of the FAB which were included were Facial Affect Discrimination, and Facial Affect Naming. In the facial affect discrimination subtest participants must determine whether two faces depict the same or different emotional expressions. In the Facial Affect Naming subtest, participants is asked to name the emotion depicted by each face (i.e., happy, sad, angry, fear, neutral). Test–retest reliability of the FAB ranges from 0.89–0.97.

(3) The Interpersonal Reactivity Index (IRI)

The IRI [19] is a 28-item self-report instrument designed to assess empathic tendencies. The IRI consists of four separate 7-item subscales: Perspective Taking (PT), Fantasy (FS), Empathic Concern (EC), and Personal Distress (PD), which are measured using a 5-point Likert scale ranging from “Does not describe me well” to “Describes me very well”. PT refers to the tendency to spontaneously adopt the psychological point of view of others. FS describes the likelihood that a person identifies with a fictional character. EC assesses individuals’ feelings of concern and compassion for others. Lastly, PD indicates the extent that a person feels uneasiness or worry when exposed to the negative experience of others. The IRI has robust validity and is among the most widely used measures of empathy [20].

(4) The Personality Assessment Inventory (PAI)

The Personality Assessment Inventory (PAI) is a self-administered, objective test of personality and psychopathology designed to provide information on critical client variables in behavioural health settings [21]. The PAI is a 344-item questionnaire in which there are 22 non-overlapping subscales. The primary PAI outcome for this study was the ‘Borderline Features’ scale (BOR), and its subscales which focuses on symptoms and traits indicative of a BPD, i.e., affective instability (BOR A), identity problems (BOR I), negative relationships (BOR N), self-harm (BOR S). The BOR consists of 24 items with each subscale containing 6 items. The respondent is asked to check one of four response options indicating the extent to which the item statement accurately describes them. For each scale responses are standardized with reference to a national census-matched sample of community adults. The PAI has robust content and discriminant validity as well as internal consistency reliability estimates [22,23].

(5) Narcissistic Personality Inventory-16

The Narcissistic Personality Inventory-16 (NPI-16) is a 16-item unidimensional measure of subclinical narcissism [24]. The NPI-16 is a shortened version of the Narcissistic Personality Inventory-40 and derives its items from that measure. Items are presented in pairs of statements, e.g., “I don’t mind following orders” and “I like having authority over people” and participants mark the statement which they agree with the most. The total score is the number of responses consistent with narcissism with a total possible score of 16. Higher scores are taken as indications of higher levels of subclinical narcissism. The NPI-16 was chosen as a brief measure to measure divergent validity.

### 2.3. Statistical Analysis

Demographic characteristics and outcome data are reported as means, standard deviations, and frequencies. Based on the data obtained, classification for good internal consistency, using Cronbach’s alpha, remains at the internationally accepted value >0.70. The data was analysed using the IBM Statistical Package (International Business Machines, New York, NY, USA) for Social Sciences version 27 (SPSS v27). An alpha level of 0.05 was set for all analyses. In line with previous related research [25], an a priori power analyses for group comparisons indicated that a minimum of n = 42 would be required per group to detect a medium effect size (power = 0.8; *f* = 0.25, α = 0.05, λ = 8.0). With regard to statistical power n = 100 is the recommended minimum number of participants for a factor analysis [26], with 5 respondents per scale item also accepted [27]. Our study meets each of these three aforementioned power requirements.

Internal consistency was estimated using Cronbach’s alpha (Cronbach’s alpha ≥0.70 were considered acceptable [28,29]. Inter-item reliability was assessed using inter-item correlations with mean inter-item correlation between 0.15 and 0.50 considered good; item total corrected correlations (ITCC) >0.40 were considered acceptable [29].

Construct validity was assessed using (1) factor analysis and (2) examining associations with other assessments measuring BPD traits. A factor analysis was conducted using principal component analysis (PCA), using an oblique (oblimin) rotation. Convergent validity was estimated by calculating Pearson correlation coefficients between SI-Bord total scores and measures of the same construct, i.e., the BPD subscales of the PAI and the BOR total score. A correlation of r > 0.7 is evidence of good convergent validity [26]. Divergent validity was assessed by calculating Pearson correlation coefficients between the SI-Bord total scores and a measure of a different personality construct: NPI-16 total score. A correlation of r < 0.5 would be considered good evidence of divergent validity [29].

Participants were further dichotomized into a high and low group based on the SI-Bord total score. Low scorers were categorized as participants who scored less than or equal to the median score of the SI-Bord total score (median = 4), and high scorers were participants who scored greater than the median. A one-way ANOVA examined the difference between low/high scorers of the SI-Bord on subscales of the FAB, and the IRI. A one-way ANOVA examined the difference between the SI-Bord low/high groupings, on items of the FAB grouped based on emotional recognition valence, i.e., sad, neutral, happy, fear, and angry stimuli.

## 3. Results

The sample consisted of 151 typical community-based healthy participants from Ireland, with a self-reported ethnicity of White Irish. Participants included in this study had no comorbid physical or mental health difficulties at the time of the study, diagnosed or treated by a healthcare professional. The average duration of the experiment was approximately 25 min. The mean age of participants was 38.79 (SD = 12.37), ranging from 20 to 76. The sample was comprised of 49.67% males (*n* = 75) and 50.33% females (*n* = 76).

The internal consistency of the scale was measured, which yielded a Cronbach’s alpha at 0.71. The Mean score and Standard Deviation were normally distributed for the sample for each item on the SI-Bord, and the correlation of each item with the SI-Bord total score can be seen in Table 1.

Inter-item reliability analyses were conducted to investigate the degree to which each item related to each other, as well as the total score. This is shown in Table 2. This table highlights how of the SI-Bord constructs are quite individual to each other, yet overall contribute strongly to the scale total. The mean inter-item cross-correlation was 0.25. Each item of the SI-Bord showed an ITCC of >0.5.

A factor analysis was conducted to investigate the unitary structure of the scale. Analysis of the scree plot indicated a strong singular dimension, which explained a large proportion of variance with a marked decline between the 1st and 2nd Eigenvalues, and lesser decreases between the following eigenvalues (Eigenvalues: 2.33, 0.93, 0.77, 0.58, 0.39; reflecting each of the SI-Bord questions in order, respectively). The Kaiser-Meyer-Olkin measure of sampling adequacy was 0.717, above the recommended cut-off of 0.6. Bartlett’s test of sphericity was found to be significant (χ^2^ 2 (10) = 142.85, *p* < 0.001). The variance explained by the 1st factor, i.e., Question 1, was 46.52%.

To investigate whether the SI-Bord related to other measures of Borderline Personality Disorder Traits, several tests were employed. Concurrent validity was measured by investigating the total scores on the SI-Bord, which were found to be strongly significantly positively correlated with total scores on BOR-A (r = 0.60; *p* < 0.001), BOR-I (r = 0.67; *p* < 0.001), BOR-N (r = 0.61; *p* < 0.001), PAI Borderline Total (r = 0.76; *p* < 0.001) and moderately significantly positively correlated with BOR-S (r = 0.39; *p* < 0.001). In terms of divergent validity, the total score on the SI-Bord was not found to be significantly correlated with total score on the NPI-16 (r = 0.131; *p* = 0.11); this relationship is similar to that of the PAI Borderline total and the NPI-16 (r = 0.036; *p* = 0.660).

Participants were stratified, as outlined above, into lower and higher SI-Bord scoring groups. The overall performance on measures of social cognition, i.e., emotion discrimination, emotion recognition, and interpersonal reactivity can be seen in Table 3. Significant differences between groups were observed for performance on the total correct score of the emotion recognition task, and individuals within the high SI-Bord group also self-reported significantly higher levels of personal distress. A one-way ANOVA was performed to investigate specific differences between high/low SI-Bord scorers on the individual emotional stimuli of the FAB. Analysis found a significant difference between high and low scorers on the Sad (F(1,149) = [6.59], *p* = 0.01) and Neutral stimuli (F(1,149) = [12.0], *p* = 0.001), though no significant effect was found for the Happy (F(1,149) = [0.51], *p* = 0.48), Fearful (F(1,149) = [0.01], *p* = 0.92), and Angry stimuli (F(1,149) = [0.15], *p* = 0.70).

## 4. Discussion

This study aimed to investigate the psychometric properties of the SI-Bord in a group of 151 community-based typical controls, as the SI-Bord was initial developed and validated with within a university population. Additionally, it examined the relationship between self-report on the SI-Bord and measures of social cognition, in comparison to well-validated measure of BPD traits. The SI-Bord was found to display good reliability and validity, with good convergence and divergence with other measures.

The measure showed good reliability as evidenced by acceptable internal consistency with a Cronbach’s alpha of 0.71 and good inter-item reliability with a mean inter-item correlation coefficient of 0.25 and with each item scoring an ITCC of >5. While this Cronbach’s alpha is acceptable, it is on the lower end of acceptability. This may be due to the small number of items within the measure. This Cronbach’s alpha while slightly lower than that reported in previous studies [17], is consistent with a trend of acceptable internal consistency. Likewise, this is comparable to the Cronbach’s alpha of 0.87 found in a non-clinical sample of the borderline scale of the PAI [30]. Additionally, the mean inter-item correlation is comparable to the 0.22 reported on the borderline scale of the PAI. The SI-Bord showed good validity as shown by good construct, convergent, and divergent validity. Construct validity was measured through PCA with a KMO above the recommended cut-off of 0.6. Good concurrent validity was found through moderate-strong significantly positive correlations with other measures of BPD symptomology Good divergent validity was found through no significant association with the SI-Bord and measures of different constructs. While there is ongoing debate around the clinical classification of BPD as a distinct mental health diagnosis [31], this research would suggest that the SI-Bord is a valid tool for investigating traits associated with BPD, in line with its original validation [17].

Given the evidence of previous research that people with BPD tend to score lower on measures of social cognition than non-clinical populations [32], it was decided to investigate whether people who scored higher on the SI-Bord would perform worse on measures of social cognition. The SI-Bord was found to show significant differences between low and high SI-Bord scorers on subscales of the social cognition measures FAB, and IRI. While there were non-significant differences for the other subscales of the FAB, and IRI, there was a general trend of higher scorers on the SI-Bord scoring higher in the direction of BPD symptomology for each subscale tested. An additional investigation into whether the emotional valence of the FAB stimuli was more sensitive to differences found a significant difference between high and low scorers of the SI-Bord on the sad and neutrally valanced stimuli. This finding is consistent with previous research which proposes that the social cognition deficit in BPD centres around recognition of neutral or ambiguous facial expressions [32,33,34]. No significant effect was found for the happy, fearful, and angry stimuli. While some postulate that the deficit in social cognition stems from difficulty distinguishing neutrally valanced expressions, others point to rather the lack of a negatively valanced marker which causes difficulty [35]. Likewise, previous research has found no differences between people with BPD and those without, with some even finding the BPD group to outperform the non-BPD group on measures of social cognition [36]. These conflicting findings are also present in this study’s finding. While these findings support evidence for a particular impairment in identifying neutral stimuli, they also highlight the multicomponent aspects of social cognition and dissociable processes involved.

### Strengths and Limitations

This study has strengths and limitations which future research can continue to build on. For example, participants completed self-report measures of personality traits, and future research may employ a clinician-led interview process. This could further be done alongside previously mentioned interview-based measures of BPD traits to investigate similarities and differences on the SI-Bord. Secondly, both a strength and limitation of the current study is the community-based sample. While this study builds on the current literature on student samples and includes measures of social cognition, a limitation is that discrimination against those with and without a diagnosis of BPD could not be considered. Thirdly, future research may employ additional measures of social cognition, both from a domain-based approach, i.e., inclusion of cognitive theory of mind and other facets not tested here, as well as within the same domain, i.e., emotion recognition from another assessment to validate findings in a larger cohort. While this work primarily related to the investigation of psychometric properties of the SI-Bord in a community-based sample, it highlights the need for further work in social cognitive phenotyping and personality traits.

## 5. Conclusions

In accordance with previous research [17,30], the results of this study support the reliability and validity of the SI-Bord in a non-clinical community sample of healthy participants. Higher outcomes on the measure were shown to relate to higher levels of self-reported personal distress, and lower outcomes on emotion recognition. Within the emotion recognition test, the largest significant between group difference was observed for sad stimuli, though further research with larger samples and multiple tests paradigms are required to replicate these findings. The speed at which people responded accurately was not measured in this study, nor were broader cognitive functions such as executive function, which may be an avenue for future research. Reports on the SI-Bord did not relate otherwise to subscales on the interpersonal reactivity index, or the processes involved in discriminating between different emotions. Overall, the SI-Bord is shown to be a reliable and valid measure of BPD traits which may be incorporated into research designs, and future further clinical validation is needed.

## Figures and Tables

**Table 1:** Mean (M) and standard deviation (SD) of each item of the SI-Bord (N = 151).

	SI-Bord Items	Range	M	SD	r
1	When people with ties to me leave me, I can barely live	(0–3)	0.867	0.838	0.588 **
2	The relationship between me and those I am bound to fluctuate between when good is very good and when bad is very bad	(0–3)	0.953	0.947	0.658 **
3	My feelings suddenly change, such as “I don’t know who I am”, “I don’t know where I am going”, or “I feel lonely”, “I have no goals”	(0–3)	1.24	1.10	0.779 **
4	I threaten to hurt myself or attempt to hurt myself or have attempted suicide	(0–3)	0.278	0.654	0.595 **
5	My mood changes suddenly, for example, from normal to irritability, depression, or anxiety	(0–3)	1.19	0.957	0.782 **
	SI-Bord Total	(0–15)	4.53	3.08	

Note: r represents each respective item’s correlation to the total SI-Bord score; ** *p* < 0.005.

**Table 2:** Inter-item cross-correlation and SI-Bord total score.

		Q1	Q2	Q3	Q4	Q5
Q1	When people with ties to me leave me, I can barely live	-	0.236	0.216	0.335	0.215
Q2	The relationship between me and those I am bound to fluctuate between when good is very good and when bad is very bad	0.236	-	0.337	0.193	0.407
Q3	My feelings suddenly change, such as “I don’t know who I am”, “I don’t know where I am going”, or “I feel lonely”, “I have no goals”	0.216	0.337	-	0.349	0.600
Q4	I threaten to hurt myself or attempt to hurt myself or have attempted suicide	0.335	0.193	0.349	-	0.350
Q5	My mood changes suddenly, for example, from normal to irritability, depression, or anxiety	0.215	0.407	0.600	0.350	-
	SI-Bord Total Score	0.558	0.658	0.779	0.595	0.782

**Table 3:** Descriptive statistics for the SI-Bord scorers on measures of social cognition and empathy stratified by lower SI-Bord (*n* = 82) and higher SI-Bord (*n* = 69) scorers.

Outcome Variable	SI-Bord Grouping	Mean	SD	SE	*p*	r
FAB Discrimination Correct	Lower	4.23	0.836	0.0923	0.25	−0.256 *
	Higher	4.07	0.863	0.1039		−0.176
FAB Affect Naming Correct	Lower	17.98	1.678	0.1853	0.02 *	0.053
	Higher	17.29	1.993	0.24		0.026
IRI	Lower	96.61	11.264	1.2593	0.99	0.089
	Higher	96.63	11.221	1.3607		0.059
IRI Fantasy Scale	Lower	23.46	5.002	0.5523	0.95	0.11
	Higher	23.51	4.125	0.4966		−0.033
IRI Empathic Concern	Lower	28.23	3.479	0.3889	0.11	0.005
	Higher	27.13	4.808	0.5788		−0.035
IRI Perspective taking	Lower	26.16	4.665	0.5152	0.11	−0.005
	Higher	24.93	4.675	0.567		0.036
IRI Personal Distress	Lower	18.62	4.553	0.5028	0.001 **	0.144
	Higher	21.01	4.265	0.5135		0.182

Note: SD = standard deviation; SE = standard error; * *p* < 0.05, ** *p* < 0.005. FAB = Florida Affect Battery; IRI = Interpersonal Reactivity Index

## Data Availability

The datasets used and/or analysed during the current study are available from the corresponding author on reasonable request.
